# Tissue Doppler echocardiography predicts long-term cardiovascular mortality: the Anglo-Scandinavian Cardiac Outcomes Trial (ASCOT) legacy 20-year follow-up study

**DOI:** 10.1136/openhrt-2024-002795

**Published:** 2025-02-04

**Authors:** Anenta Ratneswaren, Tong Wu, Amit Kaura, Devan Wasan, Somayeh Rostamian, Andrew Sharp, Neil R Poulter, PS Sever, Alice Stanton, Simon Thom, Darrel Francis, Alun D Hughes, Anoop SV Shah, Jamil Mayet

**Affiliations:** 1 National Heart and Lung Institute, Imperial College London, London, UK; 2 University Hospital of Wales, Cardiff, UK, Cardiff, UK; 3 Imperial College Healthcare NHS Trust, London, UK; 4 Molecular and Cellular Therapeutics, Royal College of Surgeons in Ireland, Dublin, Ireland; 5 University College London, London, UK; 6 London School of Hygiene & Tropical Medicine, London, UK

**Keywords:** Heart Failure, Diastolic, Hypertension, RISK FACTORS, Echocardiography

## Abstract

**Dear Team:**

**ABSTRACT:**

**Background:**

Left ventricular diastolic function as assessed by tissue Doppler echocardiography predicts cardiovascular event rates at 4 years of follow-up in patients with hypertension. Our aim was to evaluate whether this extends to predicting cardiovascular mortality after 20 years of follow-up.

**Methods:**

Conventional (E) and tissue Doppler (e′) echocardiography was performed on hypertensive participants in the Anglo-Scandinavian Cardiac Outcomes Trial (ASCOT) with long-term follow-up ascertained via linkage to the Office of National Statistics. Cardiovascular mortality was defined as death from coronary heart disease, stroke and other cardiovascular aetiology such as heart failure or peripheral vascular disease. Unadjusted and adjusted Cox regression survival models were constructed to investigate the association between tissue Doppler echocardiography measurements and long-term cardiovascular mortality.

**Results:**

Among 506 hypertensive patients (median age 64, interquartile range (58, 69), 87% male), there were 200 (40%) deaths over a 20-year follow-up period. 60 deaths (12%) were cardiovascular-related.

A reduction in e′ was independently associated with increased cardiovascular mortality, after adjusting for the ACC/AHA Atherosclerotic Cardiovascular Disease (ASCVD) risk score, with an inverse HR of 1.22 per 1 cm/s decrease (95% CI 1.04–1.43). A higher E/e′ ratio was independently associated with increased cardiovascular mortality, after adjusting for the ASCVD risk score, with an HR of 1.12 per 1-unit increase (95% CI, 1.02 to 1.23).

**Conclusions:**

Impaired left ventricular diastolic function, measured using tissue Doppler echocardiography through e′ and E/e′, independently predicts increased cardiovascular mortality over 20 years in hypertensive patients, highlighting its long-term prognostic significance.

**STATE OF SCIENTIFIC KNOWLEDGE**

- It is known that left ventricular diastolic function, as assessed by tissue Doppler echocardiography, predicts cardiovascular prognosis over a 5-year follow-up period. However, it is not known whether this continues to be important in the long term.

**WHAT THIS STUDY ADDS**

- Tissue Doppler echocardiography predicts cardiovascular mortality during a 20-year follow-up, independent of standard cardiovascular risk assessment. Each 1 cm/s reduction in long-axis early diastolic velocity (e′) at baseline is associated with a 22% increase in cardiovascular death.

**IMPLICATIONS OF THE PRESENT STUDY**

- This study emphasises the importance of the tissue Doppler velocity e′, which is easily measured on standard echocardiography. It offers an alternative simple measurement to enhance cardiovascular risk prediction beyond standard cardiovascular risk assessment.

## Introduction

 Left ventricular (LV) hypertrophy has long been recognised as an important example of hypertension-mediated organ damage. It predicts an increase in cardiovascular (CV) mortality and morbidity independent of traditional risk factors.^1^ However, LV hypertrophy is a relatively late consequence of hypertension.

A much earlier feature of hypertension is LV diastolic dysfunction, which has been detected in the past by evidence of left atrial enlargement on transthoracic echocardiography^2 3^ and by Doppler echocardiography assessment of transmitral flow.^4^ Each of these methods has its limitations. In particular, transmitral Doppler has the disadvantage of having a U-shaped relationship with diastolic function. The early (E) diastolic transmitral Doppler peak velocity reduces as diastolic function becomes impaired, but as diastolic function continues to worsen and filling pressure rises, there is a reversal, with an increase in the E transmitral Doppler velocity. In contrast, the late (A) diastolic transmitral Doppler peak velocity increases and then decreases as diastolic function continues to worsen.^4^ This phenomenon leads to similar patterns of transmitral Doppler flow when LV diastolic function is significantly impaired. This is termed ‘pseudonormalisation’. This pattern limits the use of transmitral Doppler in CV clinical assessment and research.

The application of tissue Doppler imaging during echocardiography provides a better tool for the assessment of LV diastolic function. Conventional Doppler imaging employs high-pass wall filters to eliminate the low-velocity and high-amplitude signals from myocardial wall motion to focus on measuring blood flow. By eliminating the high-pass filter and reducing gain amplification, tissue Doppler imaging characterises low velocity, high amplitude signals from myocardial motion only. The peak LV relaxation velocity of early filling (e′) and the ratio of the transmitral early filling velocity to peak LV relaxation velocity (E/e′) have been used to assess LV diastolic function. These markers of diastolic dysfunction do not demonstrate pseudonormalisation and have been incorporated into clinical guidelines for the assessment of diastolic function.^5 6^ Furthermore, these markers have been shown to be reproducible and reliable, with recent studies demonstrating their ability to predict adverse outcomes in hypertensive populations. For example, Sharp *et al* (2010) highlighted that the E/e′ ratio is a powerful predictor of primary cardiac events in hypertensive patients, further supporting its clinical utility in risk stratification.^7^ Furthermore, Lange *et al* (2023) emphasised that tissue Doppler parameters, including E/e′, are strongly associated with left heart remodelling in hypertensive patients and can predict long-term cardiovascular risk.^8^ These findings reinforce the value of tissue Doppler imaging as a non-invasive, reproducible method for assessing LV diastolic dysfunction, which is increasingly incorporated into clinical practice for evaluating cardiovascular risk in hypertensive patients.

The Anglo-Scandinavian Cardiac Outcomes Trial (ASCOT) was a trial of antihypertensive and statin therapy in participants with hypertension and at least three other cardiovascular risk factors.^9^ The initial participants enrolled in the ASCOT study in the UK have now been followed up over a 20-year period, including the group who were part of an echocardiography substudy. Our hypothesis was that LV diastolic function at baseline would remain predictive of CV events over this 20-year period.

## Methods

This study was approved by the South East Scotland Research Ethics Committee (18/SS/0016), the Health Research Authority Confidentiality Advisory Group (18/CAG/0044), the Independent Group Advising on the Release of Data of NHS Digital, and the Public Benefit and Privacy Panel for Health and Social Care of NHS Scotland.

A full description of ASCOT has previously been published.^9^ In brief, it was a clinical trial of antihypertensive therapy (amlodipine and perindopril vs atenolol and bendroflumethiazide) in 19 257 men and women aged 40–79 (63.0±8.5) years with hypertension. Detailed CV phenotypic data were collected on a subset of participants recruited from two centres (St Mary’s Hospital, London, UK and the Adapt Centre, Beaumont Hospital, Dublin, Ireland) as part of the Hypertension Associated Cardiovascular Disease (HACVD) substudy. A description of the substudy protocol, including quality control measures used for the acquisition of data, can be found in previous publications.^7 9^

### Study population

All 8580 ASCOT trial patients from the UK form the cohort of the ASCOT Legacy study. This study reports on the 506 patients recruited for the HACVD substudy at St Mary’s Hospital, Imperial College London. These patients were originally recruited between February 1998 and May 2000 and were flagged for mortality during long-term follow-up through the Office for National Statistics. In this report, we included all recorded deaths from recruitment up to 31 January 2019. CV mortality was defined as any death due to coronary heart disease, stroke or other CV aetiology such as heart failure and peripheral vascular disease.

### Echocardiography

Participants underwent two-dimensional and Doppler echocardiography following a 1-year period of blood pressure control in accordance with the HACVD protocol.^7^ Pulsed spectral Doppler echocardiography was performed using a 5-mm sample volume placed at the tips of the mitral leaflets parallel to inflow during diastole at end-expiration, with a sweep speed of 100 mm/s. Tissue Doppler measurements were sampled at the level of the mitral annulus with filters adjusted to obtain the lowest wall filter settings and the minimal optimal gain; Nyquist limits of 15–20 cm/s and a frame rate of 200 Hz were used. Each spectral trace was downloaded for offline analysis using the HDILab software programme by a single researcher, who was blinded to all participant outcome data and who was experienced in tissue Doppler assessment.

Peak early systolic (s′) and peak early diastolic (e′) velocities were measured at the septal and lateral walls. The averages of the septal and lateral wall velocities were calculated over three consecutive cardiac cycles. The ratio of the transmitral Doppler E wave velocity and the mean of e′ was used to calculate the E/e′ ratio.

Between echocardiographer, variability of echocardiographic measurements was assessed before the commencement of the study and subsequently at regular intervals during the study. Using the Bland–Altman method, the SD of the difference of individual variables (eg, E/e′) was found to be between 3.5 and 7.5% of the mean value, demonstrating that our reproducibility measurements were in keeping with other studies.^10 11^

### Statistical methods

The primary exposure was the tissue Doppler signal on baseline echocardiography and the primary outcome was CV mortality. We explored the association between e′, E/e′ ratio, s′, left atrial size and LV mass index and CV mortality. We also explored their associations with all-cause mortality.

Baseline patient data were summarised for those who had and did not have a CV death. They were also summarised according to the tertile of e′. Continuous data were summarised as median and IQR. Cox regression proportional hazards models were constructed to investigate the association between e′, E/e′, left atrial size and LV mass index (LVMI) with CV mortality. Both unadjusted and adjusted HRs with 95% CIs are presented. Adjustment was undertaken using the American College of Cardiology (ACC) and American Heart Association (AHA) atherosclerotic CV disease (ASCVD) risk score. The ACC/AHA ASCVD risk score is based on 10-year Pooled Cohort Equations and incorporates the effects of age, sex, ethnicity, blood pressure, lipids, smoking and diabetes.^12^

Regression models were also constructed for E/e′ and all-cause mortality. We examined the linearity of the association between e′ and the risk of CV death using Cox proportional regression models with smoothing splines for e′ values. Kaplan-Meier curves were constructed by tertile of e′.

Receiver operating characteristic (ROC) curve analysis was performed to evaluate the predictive ability of e′ and E/e′ for long-term outcomes, specifically all-cause mortality and cardiovascular mortality, over a 20-year follow-up period. The area under the curve (AUC) was calculated for each parameter to assess their discriminative power. A comparison of the AUCs between e′ and E/e′ was conducted to determine whether there were any significant differences in their predictive performance. The DeLong test was used to compare AUCs for the two parameters.

Statistical analyses were conducted using Python v3.9.13 and R v4.2.1^13^ in RStudio for Windows.^14^

## Results

### Baseline characteristics

Demographic data for the cohort are presented in table 1 according to CV status. They are presented according to the tertile of e′ in online supplemental table 1. The median follow-up period was 19 (IQR 13 to 20) years. Among the 506 participants, 200 participants died, of whom 60 died of cardiovascular disease. Participants who suffered a CV death were older and more likely to have diabetes than those who did not. The echocardiographic data are presented in table 2. The differences in echocardiographic parameters between those who experienced CV death and those who did not were most markedly significant for E/e′ and e′

**Table 1:** Baseline demographic data by cardiovascular death

	No cardiovascular death	Cardiovascular death	P value
Number of participants	446	60	
Age (median (IQR))	63.3 (58, 68.3)	68.4 (63.3, 73.3)	<0.001
Sex, male (%)	394 (88.3)	48 (80.0)	0.106
Ethnicity (%)			0.850
White/European	343 (76.9)	49 (81.7)	
African-Caribbean	48 (10.8)	6 (10.0)	
South Asian	35 (7.8)	4 (6.7)	
Mixed/other	20 (4.5)	1 (1.7)	
Smoker (%)	99 (22.2)	13 (21.7)	1.000
Known diabetes (%)	106 (23.8)	22 (36.7)	0.046
Known vascular disease (%)	48 (10.8)	12 (20.0)	0.062
BMI, kg/m^2^ (median (IQR))	28.1 (25.4, 30.7)	28.1 (25.7, 30.5)	0.859
Systolic BP, mm Hg (median (IQR))	151.5 (144, 165.9)	156.8 (145.9, 170.9)	0.079
Diastolic BP, mm Hg (median (IQR))	92 (87, 96.9)	91.3 (85, 96.3)	0.339
Glucose, mmol/L (median (IQR))	5.4 (5.0, 6.1)	5.7 (5.2, 6.6)	0.028
Creatinine, μmol/L (median (IQR))	99 (89, 111)	101 (91, 111)	0.816
Cholesterol, mmol/L (median (IQR))	5.7 (5.1, 6.3)	6.0 (5.1, 6.7)	0.253
HDL cholesterol, mmol/L (median (IQR))	1.3 (1.1, 1.5)	1.2 (1.0, 1.5)	0.102
LDL cholesterol, mmol/L (median (IQR))	3.6 (3.1, 4.3)	3.7 (3.0, 4.6)	0.274
Triglycerides, mmol/L (median (IQR))	1.5 (1.1, 2.0)	1.8 (1.1, 2.3)	0.073
On antihypertensives (%)	425 (95)	60 (100)	0.170
On lipid lowering therapy at baseline (%)	50 (11)	8 (13)	0.788
On aspirin (%)	108 (24)	18 (30)	0.416

**Table 2:** Echocardiographic results

Echocardiographic data	No CV death	CV death	P value
Participant number	446	60	
E/e′ (median (IQR))	7.5 (6.3, 9.0)	8.6 (7.1, 10.9)	0.001
e′, cm/s (median (IQR))	8.4 (7.2, 9.7)	7.5 (6.4, 8.9)	0.001
Transmitral E wave velocity, cm/s (median (IQR))	62.6 (55.3, 72.4)	64.2 (54.9, 77.1)	0.272
LV mass, g (median (IQR))	213.9 (176.0, 261.8)	214.5 (182, 252.4)	0.706
LV mass (indexed), g/m^2^ (median (IQR))	109.4 (92.1, 131.2)	111.3 (99.3, 134)	0.496
Interventricular septal thickness (median (IQR))	1.2 (1.1, 1.4)	1.3 (1.2, 1.4)	0.058
Left atrial diameter, cm (median (IQR))	4.1 (3.8, 4.6)	4.3 (4.0, 4.6)	0.285
Ejection fraction, % (median (IQR))	69.0 (59.8, 78.0)	69.6 (57.0, 77.1)	0.706
Mean tissue Doppler s′ velocity, cm/s (median (IQR))	9.4 (8.0, 10.9)	9.2 (7.8, 10.9)	0.511
Transmitral E/A ratio (median (IQR))	9 (0.7, 1.0)	0.8 (0.7, 0.9)	0.015

### Survival analysis

**Figure 1:**
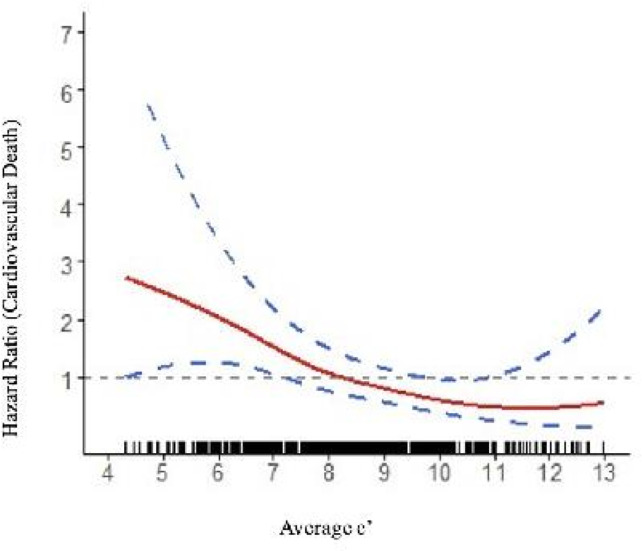
Unadjusted association between e′ (the peak early LV relaxation velocity) and the HR for cardiovascular death. An inverse relationship was observed; the lower e′, the higher the predicted CV mortality during the 20-year follow-up. Dotted curves represent pointwise 95% confidence limits. The reference for the HR was 1, at the median e′ value of 8.1 cm/s. Small black lines (rug plot) at the bottom indicate the distribution of individual data points along the x-axis, helping to visualise data density.

Tables3^6^ show the Cox regression analyses. For each decrease of 1 cm/s of e′, the unadjusted HR was 1.32 (95% CI 1.14 to 1.54) and for each point increase of E/e′, the unadjusted HR was 1.17 (95% CI 1.08 to 1.27). These remained significant when adjusted for ACC/AHA ASCVD risk score. Neither e′ nor E/e′ were associated with all-cause mortality when adjusted. There was a near linear relationship between e′ and risk of CV death (figure 1). The ASCVD adjusted curve is similar (online supplemental figure 1).

**Table 3:** Cox proportional hazards regression analysis using e′ to predict the expected CV death HR and the expected all-cause mortality HR

E′ CV death	Hazard ratio (per 1 cm/s reduction)	CI	P value
Unadjusted	1.32	1.14 to 1.54	<0.001
Adjusted for ASCVD score	1.22	1.04 to 1.43	0.01
e′ all-cause mortality	Hazard ratio (per 1 cm/s reduction)	CI	P value
Unadjusted	1.15	1.06 to 1.25	<0.001
Adjusted for ASCVD score	1.06	0.98 to 1.16	0.12

**Table 4:** Cox proportional hazards regression analysis using E/e′ to predict the expected CV death HR and the expected all-cause mortality HR

E/e′ CV death	Hazard ratio (per increase in 1 unit)	CI	P value
Unadjusted	1.17	1.08 to 1.27	<0.001
Adjusted for ASCVD score	1.12	1.02 to 1.23	0.01
E/e′ all-cause mortality	HR (per increase in 1 unit)	CI	P value
Unadjusted	1.06	1.00 to 1.12	0.05
Adjusted for ASCVD score	1.01	0.95 to 1.07	0.74

**Table 5:** Cox proportional hazards regression analysis using left atrial diameter to predict the expected CV death HR and the expected all-cause mortality HR

Left atrial diameterCV death	HR(per increase in 1 cm)	CI	P value
Unadjusted	1.23	0.79 to 1.89	0.356
Adjusted for ASCVD score	1.12	0.73 to 1.71	0.601
Left atrial diameterAll-cause mortality	HR(per increase in 1 cm)	CI	P value
Unadjusted	1.06	0.84 to 1.35	0.62
Adjusted for ASCVD score	1.00	0.79 to 1.26	0.978

**Table 6:** Cox proportional hazards regression analysis using LVMI to predict the expected CV death HR and the expected all-cause mortality HR

LVMICV death	HR	CI	P value
Unadjusted	1.00	0.99 to 1.01	0.633
Adjusted for ASCVD score	1.00	0.99 to 1.01	0.873
LVMIAll-cause mortality	HR	CI	P value
Unadjusted	1.00	1.00 to 1.01	0.418
Adjusted for ASCVD score	1.00	1.00 to 1.01	0.787

Traditional echocardiographic measures of cardiac target organ damage, such as left atrial diameter and LVMI, did not predict CV mortality in this population (tables3^6^). The tissue Doppler peak systolic velocity, s′, also did not predict CV death.

**Figure 2:**
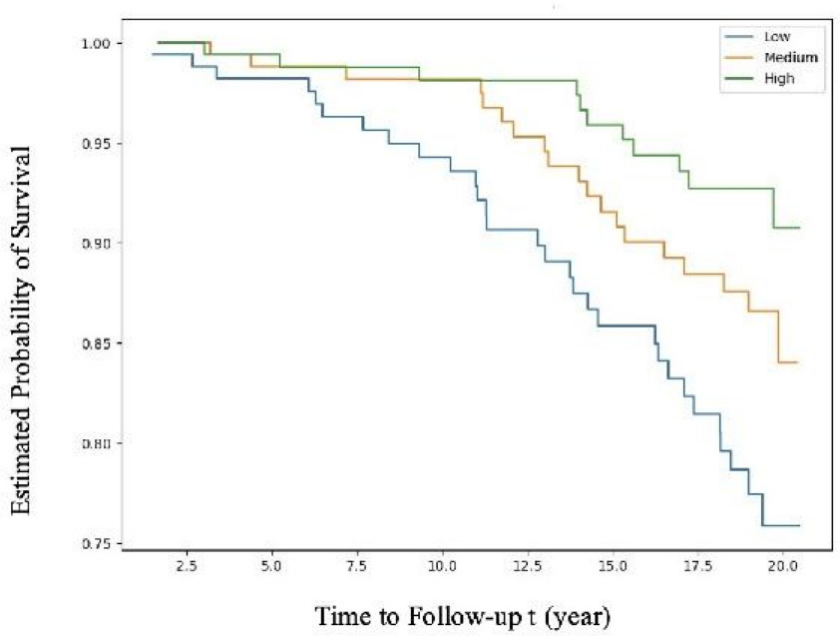
Kaplan-Meier survival curves for cardiovascular mortality by tertiles of average e′ values. These are presented as low (e′ 4.32–7.52) in blue, medium (e′ 7.52–9.19) in orange and high (e′ 9.19–14.86) in green. The highest risk tertile had more than twice the CV death rate of the lowest tertile over 20 years of follow-up.

The Kaplan-Meier cardiovascular survival analyses for participants in this cohort divided by tertiles of e′ are presented in figure 2. The average e′ values were divided into three equal groups for Kaplan-Meier survival analysis (low (4.32–7.52) in blue, medium (7.52, 9.19) in orange and high (9.19, 14.86) in green). A similar plot for E/e′ can be found in online supplemental figure 2.

During follow-up, there were observable between-group differences in CV mortality. The highest risk tertile (17.8% death rate) had more than twice the CV death rate of the lowest tertile (6.5% death rate). The log-rank test suggests that the difference is significant (p=0.002).

We conducted ROC curve analyses to compare the predictive ability of e′ and E/e′ for both cardiovascular mortality and all-cause mortality over the 20-year follow-up period. For cardiovascular mortality, the AUC for e′ was 0.64 (95% CI 0.56 to 0.71), while the AUC for E/e′ was 0.63 (95% CI 0.55 to 0.71). These results show no significant difference in the AUC between e′ and E/e′ (p=0.82), suggesting that both parameters have comparable predictive power for cardiovascular mortality in this cohort. A similar pattern was observed for all-cause mortality, with no significant difference in the AUC between e′ and E/e′ (p=0.18). All ROC curves are presented in online supplemental figure 3.

## Discussion

The main finding of this study was that standard tissue Doppler parameters of LV diastolic function, e′ and E/e′, were predictive of CV mortality over 20 years of follow-up. The predictive ability of both e′ and E/e′ remained significant after adjustment for the ACC/AHA ASCVD risk score, which includes traditional CV risk factors. For each unit decrease of e′, the increase in risk of CV death was 22%, and for each unit increase of E/e′, the increased risk was 12% on adjusted analyses.

Tissue Doppler-derived measurements have become a recommended part of the echo assessment of LV diastolic function and are suggested in standard clinical guidelines. These advise that e′ peak velocity is measured at both the septal annulus and lateral wall annulus and is averaged. This was the measurement made in the present study, emphasising the importance of including these in the standard echocardiographic examination. The e′ peak velocity is relatively independent of loading conditions and is a reproducible measurement.^6^

### Why were LV ejection fraction and LV hypertrophy not predictive?

This group of participants had only a moderate CV risk at baseline (hypertension and three additional risk factors, two of which could have been male sex and age over 55). They had a normal LV ejection fraction, and the majority had a normal LV mass index. Although an abnormal ejection fraction is known to be associated with a worse prognosis, there is no evidence that LV ejection fraction predicts prognosis within the normal range and these results are in keeping with previous studies. There is evidence that LV hypertrophy is a strong predictor of an increased CV risk, independent of traditional risk factors.^15 16^ We did not observe this within this cohort of patients and it may be related to the relatively narrow range of baseline LV mass. There was a trend toward left atrial diameter being predictive of cardiovascular mortality, but this was not statistically significant. The absence of risk prediction of traditional cardiac markers in this cohort emphasises the strong predictive ability of the tissue Doppler parameters. Our findings suggest that LV diastolic dysfunction assessment using e′ or E/e′ are more sensitive predictors of an adverse CV outcome compared with LV mass. Tissue Doppler e′ and E/e′ are easier to measure and more reproducible than LV ejection fraction and LV mass.^16^

### Why might E’ and E/E’ be predictive of CV outcomes?

Studies of patients with pathological left ventricular hypertrophy have described a strong relationship between LV mass and cardiovascular events.^1 16^ The hypertrophic process leads to myocyte hypertrophy and an increase in cardiac fibrosis, with the latter thought to be the key link with adverse cardiovascular events.^17^

A significant relationship has been demonstrated between e′ and τ, the time constant of LV relaxation in human studies.^4 18^ It is known that LV relaxation and e′ worsen with age, and these are likely due to an increase in LV fibrosis.^19^ Human studies show a strong correlation between e′ and interstitial fibrosis.^20^ The strong relationship between e′ and cardiovascular death may be because it is a better marker than LV mass or s′ of fibrosis. Both e′ and s′ decrease with age but the inverse relationship between e′ and age has a much steeper slope, supporting this hypothesis.

Pathologies that are known to cause premature coronary disease and cerebrovascular disease, such as hypertension, diabetes and coronary disease, also cause cardiac fibrosis, and this may be a common pathway leading to an increased risk of heart failure or arrhythmic cardiac death. Vascular wall changes occur in parallel with cardiac changes, and a lower e′ may also be a marker for a more severe fibrosis in arterial walls, leading to vascular stiffening and an increased risk of plaque rupture.^21^,^24^

Research has shown that arterial stiffness is strongly associated with diastolic dysfunction, with higher arterial stiffness linked to worse LV relaxation and elevated E/e′ ratios.^25^ This relationship is particularly significant in conditions like heart failure with preserved ejection fraction (HFpEF) and atrial fibrillation (AF), where combined reductions in both e′ and s′ velocities are predictive of a 12-fold increase in adverse clinical outcomes, including heart failure decompensation, ischaemic stroke and cardiovascular death.^26^ Additionally, lifestyle factors such as obesity, hypertension and physical inactivity contribute to systemic inflammation, which in turn drives myocardial fibrosis by promoting fibroblast activation and collagen deposition, further worsening diastolic dysfunction. Addressing these modifiable lifestyle factors is critical for preventing and managing diastolic dysfunction.^27^

### Might tissue Doppler echocardiography have a role in CV risk assessment?

Although this cohort of participants had a high event rate after 20 years of follow-up (40% overall mortality, 12% cardiovascular mortality), it is important to emphasise that at baseline, these participants were at intermediate risk over the initial follow-up period; the very high event rates are a consequence of the very long follow-up in the study. International guidance at present does not make firm recommendations on the inclusion of biomarkers as risk modifiers in cardiovascular risk estimation.^28^ However, international guidance does indicate consideration of these modifiers, especially for individuals who remain close to a decision threshold for preventative pharmacotherapy (ie, statin therapy for those at intermediate risk). This includes the potential use of coronary CT, either in the form of coronary artery calcium scoring or CT coronary angiography, to improve risk assessment. This is particularly relevant to participants who remain reluctant or uncertain over whether to take statin therapy. It is also important to note there has been no prospective study supporting the use of coronary CT risk assessment for improved cardiovascular risk management and current guidelines highlight feasibility and expense as major limitations to widespread adoption.^28^ In the multiethnic study of atherosclerosis study, LV hypertrophy was a stronger predictor than coronary artery calcium scoring at predicting CV mortality,^29^ but LV mass is difficult to measure in routine clinical practice accurately and reproducibly. Tissue Doppler echo e′, which appears to be a better risk predictor than LV mass, is easy to measure and more reproducible and may provide an alternative to coronary CT scanning for enhanced CV risk assessment in the intermediate risk group.^30 31^ Our findings suggest that the predictive ability of tissue Doppler echocardiography extends to 20 years. Importantly, current standard transthoracic echocardiography includes tissue Doppler measurement of diastolic function, providing a low-cost assessment. Moreover, echocardiography does not involve ionising radiation. Since e′ might be considered a marker of cardiovascular ageing due to fibrosis, this parameter might be used to describe ‘heart age’ to individuals. The use of heart age as a concept for cardiovascular risk has been shown to have value for behaviour modification.^32^

We observed a continuous relationship between e′ values and cardiovascular mortality. This finding indicates that there is no clear threshold for e′ but rather a progressive increase in risk as e′ values decline. As such, pinpointing a specific cut-off value for e′ that can be considered clinically significant is difficult. Rather than relying on a single threshold, e′ should be interpreted alongside other clinical metrics and relevant comorbidities, to provide a more comprehensive assessment of cardiovascular risk. This integrated approach will help clinicians make more informed decisions regarding patient care.

### Study limitations

The ASCOT study recruited participants with hypertension and at least three other CV risk factors. Participants had close monitoring and a strict blood pressure target for the duration of the main study. Such exemplary treatment is uncommon outside the context of a clinical trial. Echocardiograms in this study were done in the late 1990s, so some of the more recent echocardiographic measurements of LV function, such as global longitudinal strain, were unavailable in this cohort. Like many clinical studies, the majority of participants were male, aged >60 years, and predominantly of white European ethnicity. This should prompt caution when extrapolating the findings to other groups. To enhance the generalisability of these results, future research should aim to replicate these findings in more diverse populations, including women, individuals from different ethnic backgrounds, and younger patients.

## Conclusion

Tissue Doppler e′ and E/e′ independently predict 20-year CV mortality in a hypertensive population and outperformed traditional echocardiographic measures. Tissue Doppler echocardiography provides additional long-term prognostic information over and above that of traditional clinical CV risk assessment.

## supplementary material

10.1136/openhrt-2024-002795 online supplemental file 1

## Data Availability

Data are available upon reasonable request.
