# Supplementary material for: Tissue Doppler echocardiography predicts long-term cardiovascular mortality: the Anglo-Scandinavian Cardiac Outcomes Trial (ASCOT) legacy 20-year follow-up study
Source: Open Heart. 2025 Feb 4;12(1):e002795. doi: 10.1136/openhrt-2024-002795 (PMC11795408; doi:10.1136/openhrt-2024-002795)
Supplement: online supplemental file 1 [file openhrt-12-1-s001.docx]

**SUPPLEMENTAL APPENDIX:**

**Supplemental Table 1. Baseline demographic data by tertile of e’**

| e’ | Low | Medium | High | p |
| --- | --- | --- | --- | --- |
| Number of participants | 169 | 168 | 169 |  |
| Age (median [IQR]) | 66.3 [61.7, 71.1] | 63.6 [58.4, 68.3] | 61.2 [55.5, 66.5] | <0.001 |
| Sex = Male (%) | 143 (84.6) | 147 (87.5) | 152 (89.9) | 0.337 |
| Ethnicity (%) |  |  |  | 0.002 |
| White/European | 123 (72.8) | 126 (75.0) | 143 (84.6) |  |
| African | 27 (16.0) | 20 (11.9) | 7 (4.1) |  |
| South Asian | 7 (4.1) | 17 (10.1) | 15 (8.9) |  |
| Mixed/Other | 12 (7.1) | 5 (3.0) | 4 (2.4) |  |
| Smoker (%) | 26 (15.4) | 40 (23.8) | 46 (27.2) | 0.026 |
| Known diabetes (%) | 46 (27.2) | 48 (28.6) | 34 (20.1) | 0.159 |
| Known vascular disease (%) | 20 (11.8) | 22 (13.1) | 18 (10.7) | 0.786 |
| BMI, kg/m2 (median [IQR]) | 27.8 [25.7, 30.2] | 28.7 [25.2, 30.9] | 28.1 [25.4, 31.2] | 0.724 |
| Systolic BP, mmHg (median [IQR]) | 152.5 [144.5, 168.0] | 152.8 [144.4,166.1] | 151.0 [144.0, 165.0] | 0.469 |
| Diastolic BP, mmHg (median [IQR]) | 90.5 [86.0, 97.0] | 92.0 [86.9, 96.5] | 93.0 [88.0, 97.5] | 0.263 |
| Glucose, mmol/L (median [IQR]) | 5.4 [5.0, 6.4] | 5.4 [5.0, 6.2] | 5.4 [5.0, 6.0] | 0.606 |
| Creatinine, umol/L (median [IQR]) | 100.50 [90.25, 114.75] | 98.00 [89.00, 109.00] | 98.00 [88.00, 108.00] | 0.117 |
| Cholesterol, mmol/L (median [IQR]) | 5.7 [5.1, 6.3] | 5.7 [4.9, 6.3] | 5.8 [5.1, 6.5] | 0.452 |
| HDL Cholesterol, mmol/L (median [IQR]) | 1.3 [1.1, 1.6] | 1.3 [1.1, 1.5] | 1.3 [1.0, 1.5] | 0.658 |
| LDL Choletserol, mmol/L (median [IQR]) | 3.6 [3.0, 4.3] | 3.6 [3.0, 4.2] | 3.7 [3.2, 4.4] | 0.600 |
| Triglycerides, mmol/L (median [IQR]) | 1.4 [1.1, 1.9] | 1.5 [1.0, 2.0] | 1.6 [1.1, 2.0] | 0.418 |
| On antihypertensives (%) | 166 (98.2) | 158 (94.0) | 161 (95.3) | 0.141 |
| On lipid lowering therapy (%) | 22 (13.0) | 17 (10.1) | 19 (11.2) | 0.701 |
| On aspirin (%) | 53 (31.4) | 42 (25.0) | 31 (18.3) | 0.022 |
| CVD death (20-years) | 30 (17.8) | 19 (11.3) | 11 (6.5) | 0.006 |
| All cause death (20-years) | 85 (50.3) | 61 (36.3) | 54 (32.0) | 0.002 |

**Supplementary *Figure 1. Adjusted association between e’ (the peak early LV relaxation velocity) and the hazard ratio for cardiovascular death.***

*Adjustment for ACC/AHA ASCVD risk score. An inverse relationship was observed; the lower e’, the higher the predicted CV mortality during the 20-year follow-up. Dotted curves represent pointwise 95% confidence limits. The reference for the hazard ratio was 1, at the median e’ value of 8.1 cm/s. Small black lines (rug plot) at the bottom indicate the distribution of individual data points along the x-axis, helping to visualize data density.*


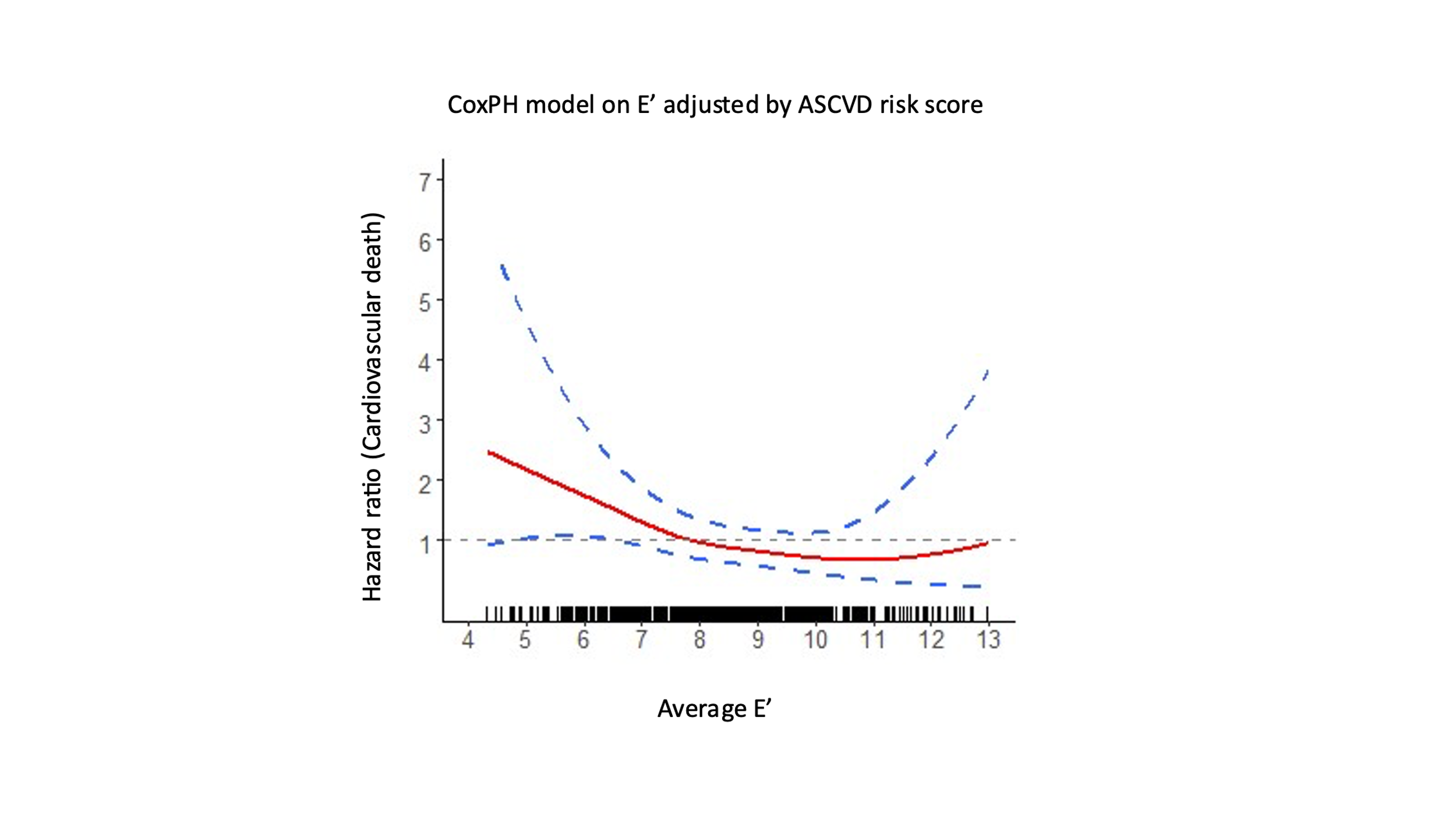


Average e’

Hazard Ratio (Cardiovascular Death)

**Supplementary Figure 2. *Kaplan-Meier survival curves for cardiovascular mortality by tertiles of average E/e' values.***

*These are presented as low (E/e’ 3.9 – 6.8) in blue, medium (E/e’ 6.8 – 8.6) in orange and high (E/e’ 8.6 – 21.3) in green.*


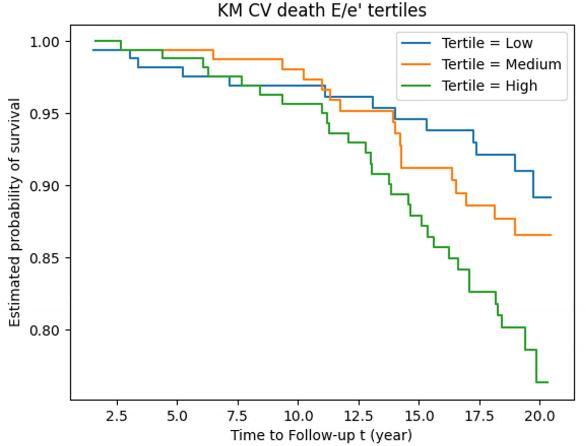


**Supplementary Figure 3. Receiver operating characteristic (ROC) curves for e' (black) and E/e' (blue) in predicting cardiovascular mortality (left) and all-cause mortality (right) over 20 years of follow-up**

| 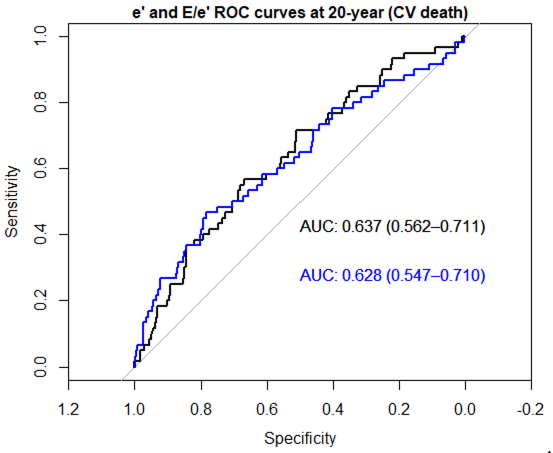 | 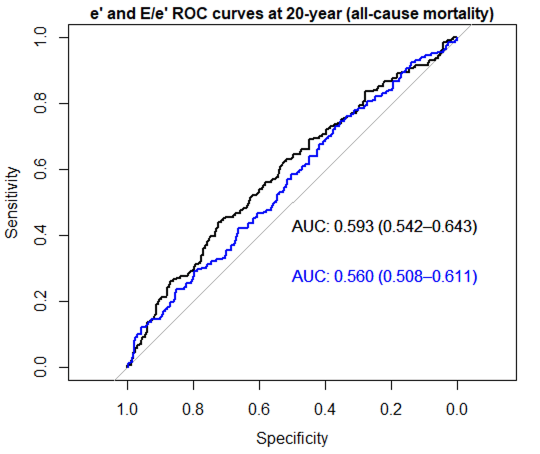 |
| --- | --- |
